# Extracellular Vesicles Contribute to Mixed-Fungal Species Competition during Biofilm Initiation

**DOI:** 10.1128/mbio.02988-22

**Published:** 2022-11-15

**Authors:** Robert Zarnowski, Justin Massey, Aaron P. Mitchell, David Andes

**Affiliations:** a Department of Medicine, University of Wisconsin-Madison, Madison, Wisconsin, USA; b Department of Microbiology and Immunology, University of Wisconsin-Madison, Madison, Wisconsin, USA; c Department of Microbiology, University of Georgia, Athens, Georgia, USA; University of Florida

**Keywords:** *Candida*, biofilms, cell adhesion, vesicles

## Abstract

Extracellular vesicles commonly modulate interactions among cellular communities. Recent studies demonstrate that biofilm maturation features, including matrix production, drug resistance, and dispersion, require the delivery of a core protein and carbohydrate vesicle cargo in *Candida* species. The function of the vesicle cargo for these advanced-phase biofilm characteristics appears to be conserved across *Candida* species. Mixed-species interactions in mature biofilms indicate that vesicle cargo serves a cooperative role in preserving the community. Here, we define the function of biofilm-associated vesicles for biofilm initiation both within and among five species across the *Candida* genus. We found similar vesicle cargo functions for several conserved proteins across species, based on the behavior of mutants. Repletion of the adhesion environment with wild-type vesicles returned the community phenotype toward reference levels in intraspecies experiments. However, cross-species vesicle complementation did not restore the wild-type biology and in fact drove the phenotype in the opposite direction for most cross-species interactions. Further study of mixed-species biofilm adhesion and exogenous wild-type vesicle administration similarly demonstrated competitive interactions. Our studies indicate that similar vesicle cargoes contribute to biofilm initiation. However, vesicles from disparate species serve an interference competitive role in mixed-*Candida* species scenarios.

## OBSERVATION

Biofilm formation is a common growth state for most microbes (1–3). Microbe adhesion to a surface marks biofilm initiation, which is followed by cell proliferation, production of and community encasement in an extracellular matrix, and eventually dispersion of cells from the mature cell population (4).

While biofilms can exist in a monophyletic state, they often flourish in mixed-species communities that include both intrakingdom and interkingdom relationships (5–8). These cell populations are dynamic, with extensive interactions among their residents and the environment. Interactions within these structured assemblies can be positive, negative, or neutral. These relationships shape community behavior (9–13). Microbial interactions within a biofilm can involve a multitude of mechanisms, including physical, metabolic, and chemical signaling. For example, Candida albicans cells exhibit cooperative behavior with Staphylococcus aureus via production of a *Candida*-produced extracellular matrix that protects both microbes from toxins and antibiotics (14). Conversely, Burkholderia thailandensis competes with other microbial species in the environment by producing antimicrobials (15).

Extracellular vesicle delivery of diverse cargo facilitates cell-to-cell communication and provides numerous cellular functions (16–20). We recently identified cooperative vesicle-driven interactions among common *Candida* species during advanced biofilm phases, including extracellular matrix production, leading to protection from antifungal therapies and dispersion from the biofilm community (21–23). Production and secretion of core polysaccharides and proteins within extracellular vesicles appear to modulate these cooperative interspecies behaviors.

The current studies explored the function of core extracellular vesicle cargo during the biofilm adhesion phase in both monomicrobic and mixed-*Candida* species communities. We found that several of the shared cargo proteins alter biofilm initiation. However, unlike the cross-species cooperative function we found for mature biofilm function, the function of non-self vesicles did not repair the adhesion mutant defect and in fact seemed to act in a competitive manner.

### Impact of core vesicle proteomes on biofilm adhesion.

**FIG 1:**
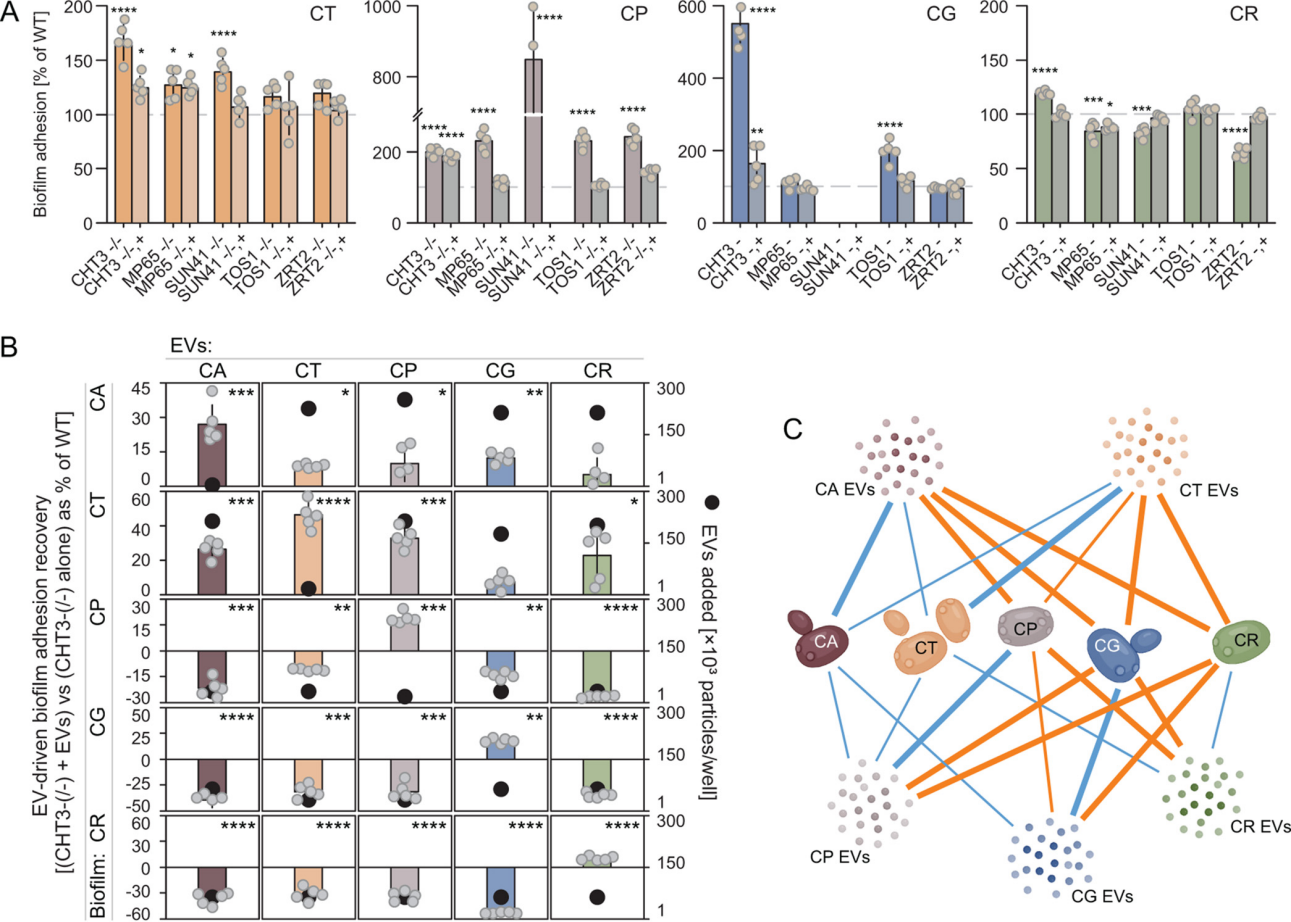
Select conserved extracellular vesicle cargo proteins are required for adhesion in early developing *Candida* biofilms. (A) Biofilm adhesion over 90 min of incubation of the *Candida* extracellular vesicle cargo core conserved protein mutants, compared to the wild-type (WT) control. Both the null deletion mutants and the corresponding complemented strains are shown. Missing bars show mutant strains that could not be genetically created. Each dot is an independent biological replicate. Data are presented as the mean ± standard deviation (SD) (*n* ≥ 5). *, *P < *0.05; **, *P < *0.01; ***, *P < *0.005; ****, *P ≤ *0.0001, using nonparametric Kruskal-Wallis one-way analysis of variance with *post hoc* uncorrected Dunn’s multiple-comparison test. (B) Effects of exogenous *Candida* biofilm extracellular vesicles (EVs) on biofilm adhesion of CHT3 null mutants, as measured by the 96-well 2,3-bis(2-methoxy-4-nitro-5-sulfophenyl)-5-carboxanilide-2*H*-tetrazolium (XTT) assay. Biofilm cultures of adhesion-dysregulated mutant strains (grouped in rows) were amended with wild-type extracellular vesicles (columns) isolated from five different *Candida* species biofilm culture supernatants. *Candida* species color-coded bars indicate a phenotypic difference between a mutant biofilm grown with and without exogenous extracellular vesicles, expressed as percent reduction of the wild-type value. Black dots indicate concentrations of exogenous extracellular vesicles required for optimal phenotypic response. (C) Network diagram summarizing extracellular vesicle-driven intrapecies and interspecies regulation of adhesion in *Candida* biofilms. A combination of positive (blue lines) and negative (orange lines) interactions between the tested *Candida* species and their extracellular vesicles were detected. The strongest phenotypic recovery was observed when a biofilm of a given species was amended with its own species-specific extracellular vesicles. CA, Candida albicans; CT, Candida tropicalis; CP, Candida parapsilosis; CG, Candida glabrata; CR, Candida auris.

We examined the impact of the five vesicle proteins present in five commonly encountered *Candida* species on the adhesion step. Three of five vesicle cargo mutants (CHT3, TOS1, and SUN41) exhibited changes in adhesion for Candida tropicalis, Candida parapsilosis, and Candida glabrata, consistent with prior studies in C. albicans (Fig. 1A; also see the supplemental methods, Table S1 and Text S1). Replacement of a wild-type allele in mutant strains resulted in an adhesion phenotype closer to that of the reference strain. Conversely, these mutants demonstrated less, but still statistically significant, impact on the Candida auris adhesion phenotype. This apparent difference in the vesicle-associated adhesion pathway is interesting, given the unique clinical colonization features of C. auris on patient skin and in the hospital environment (24–26). We speculate that this species has developed novel tools to allow biofilm initiation in these unique patient environments.

10.1128/mbio.02988-22.1 TABLE S1 *Candida* strains and genotypes utilized in the reported studies. Download Table S1, PDF file, 0.3 MB. Copyright © 2022 Zarnowski et al. 2022 Zarnowski et al. https://creativecommons.org/licenses/by/4.0/ This content is distributed under the terms of the Creative Commons Attribution 4.0 International license.

10.1128/mbio.02988-22.1 TEXT S1 Detailed methods utilized for the studies. Download Text S1, PDF file, 0.2 MB. Copyright © 2022 Zarnowski et al. 2022 Zarnowski et al. https://creativecommons.org/licenses/by/4.0/ This content is distributed under the terms of the Creative Commons Attribution 4.0 International license.

### Impact of exogenous repletion of vesicle cargo on biofilm adhesion within species.

Our previous studies examining the role of vesicle proteome cargo implicated a cargo-specific role via phenotype reversal in mutants following exogenous repletion with wild-type vesicles (containing the protein absent in the mutants) (21, 22). The current studies employed this strategy for the mutant (CHT3) that exhibited adhesion phenotypes for *Candida* species. We found that exogenous administration of wild-type extracellular vesicles to mutants within species returned the adhesion phenotype toward reference strain levels (Fig. 1B; also see the supplemental methods). This finding suggests that the presence of the protein within the extracellular vesicle is responsible for or contributes to substrate binding biology. The concentration of wild-type vesicles needed to produce this phenotypic change was relatively low, compared to vesicle concentrations produced during mature biofilm growth, but consistent with concentrations observed during the adhesion period (see Fig. S1 in the supplemental material) (21, 23).

10.1128/mbio.02988-22.3 FIG S1 Concentrations of *Candida* biofilm extracellular vesicles during early biofilm formation. Extracellular vesicles were isolated from the tested reference strains at 90 min, followed by nanoparticle tracking analysis (NTA) of filter-sterilized culture supernatants. Both qualitative and quantitative profiles of extracellular vesicles quickly undergo dramatic changes, shifting from highly convoluted heterogeneous mixtures to less complex and more abundant exosome-like nanoparticle amalgamates. Data are presented as the mean (*n* = 3). CA, Candida albicans; CT, Candida tropicalis; CP, Candida parapsilosis; CG, Candida glabrata; CR, Candida auris. Download FIG S1, PDF file, 0.2 MB. Copyright © 2022 Zarnowski et al. 2022 Zarnowski et al. https://creativecommons.org/licenses/by/4.0/ This content is distributed under the terms of the Creative Commons Attribution 4.0 International license.

### Impact of exogenous repletion of vesicle cargo on biofilm adhesion across species.

Recent studies in *Candida* species mutants found that wild-type vesicle amendment was functional across species for advanced-stage biofilm phenotypes, including matrix production, drug tolerance, and dispersion (23). For example, C. albicans cargo mutants with drug tolerance alterations were restored via administration of C. glabrata wild-type vesicles (23). For the current adhesion defects, while cargo mutant adhesion defects were returned via wild-type vesicles within species, the ability of vesicles from non-self species to return adhesion features toward the wild-type state was minimal and observed for only a few C. tropicalis-vesicle interactions. More commonly, we found that the presence of wild-type vesicles from non-self species resulted in adhesion behavior in the opposing direction (Fig. 1B and C; also see Fig. S2 and the supplemental methods). For example, providing wild-type C. parapsilosis vesicles to C. glabrata CTH3 mutants resulted in a nearly 75% change in the opposite direction, compared to administration of wild-type C. glabrata vesicles. These findings are distinct from the cooperative cross-species function observed during the mature biofilm phases, including matrix production, protection, and dispersion. The cross-species vesicle function during biofilm initiation appeared to provide a competitive function.

10.1128/mbio.02988-22.4 FIG S2 Effects of exogenous *Candida* biofilm extracellular vesicles on biofilm adhesion of CHT3 null mutants. Biofilm cultures of adhesion-altered mutant strains (grouped in rows) were amended with wild-type extracellular vesicles (columns) isolated from five different *Candida* species biofilm culture supernatants. Lines represent the mean of 8 technical replicates, and the shaded blue area represents minimal and maximal value range distribution. Marquis orange arrows indicate the concentrations of exogenous extracellular vesicles added during biofilm seeding at which maximal adhesion-altering effects were observed. Data are presented as the mean ± SD (*n* = 5). *, *P < *0.05; **, *P < *0.01; ***, *P < *0.005; ****, *P ≤ *0.0001, using nonparametric Kruskal-Wallis one-way analysis of variance with *post hoc* uncorrected Dunn’s multiple-comparison test. CA, Candida albicans; CT, Candida tropicalis; CP, Candida parapsilosis; CG, Candida glabrata; CR, Candida auris. Download FIG S2, PDF file, 0.6 MB. Copyright © 2022 Zarnowski et al. 2022 Zarnowski et al. https://creativecommons.org/licenses/by/4.0/ This content is distributed under the terms of the Creative Commons Attribution 4.0 International license.

### Mixed-*Candida* species interactions during biofilm initiation.

**FIG 2:**
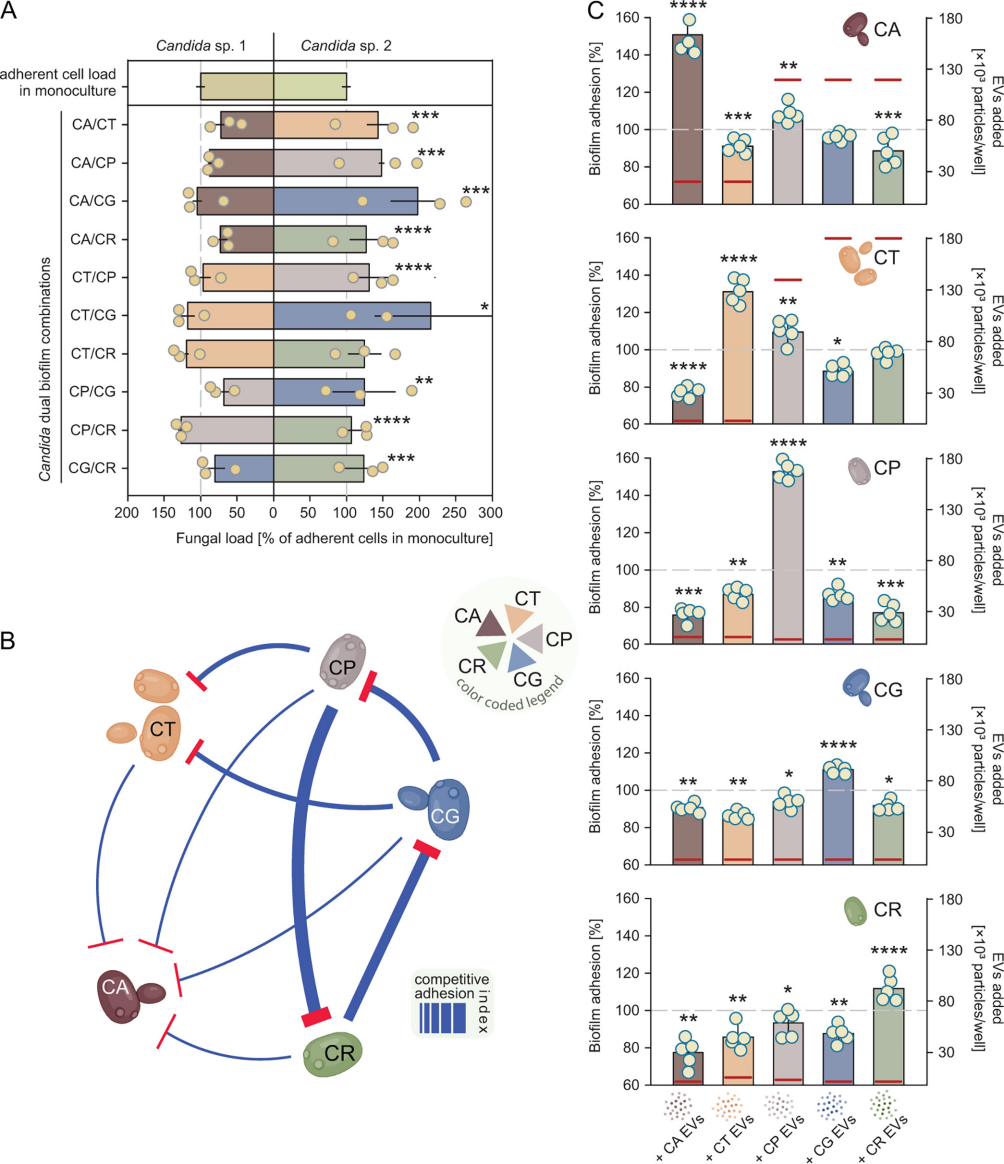
Extracellular vesicles influence the process of adhesion in early developing *Candida* biofilms. (A) Determination of adhesion competition via the *Candida* dual-species biofilm assay. Fungal cells were mixed prior to biofilm seeding. Fungal biofilm burdens were measured by qPCR using *Candida* species-specific probes. Data are presented as the mean ± SD (*n* = 4). *, *P < *0.05; **, *P < *0.01; ***, *P < *0.005; ****, *P ≤ *0.0001, using nonparametric Kruskal-Wallis one-way analysis of variance with *post hoc* uncorrected Dunn’s multiple-comparison test. Outliers were removed using the Grubbs’ test. (B) Network diagram summarizing competitive adhesion-type interactions between the tested biofilm-forming C*andida* species. T-arrows indicate the individual species that were less adherent in the mixed-species interactions. Strengths of those interactions are reflected by the thickness of the arrows, with thin lines indicating a lower competitive adhesion index and thicker arrows indicating a higher competitive adhesion index in the tested dual biofilms. Candida glabrata and Candida auris (both non-hypha-forming species), along with Candida parapsilosis, are capable of strong adherence and initially outcompete other species such as Candida albicans and Candida tropicalis. CA, Candida albicans; CT, Candida tropicalis; CP, Candida parapsilosis; CG, Candida glabrata; CR, Candida auris. (C) Effects of exogenous *Candida* biofilm extracellular vesicles (EVs) on biofilm adhesion. Biofilm cultures of wild-type *Candida* strains were amended with wild-type extracellular vesicles isolated from five different *Candida* species biofilm cultures. Extracellular vesicles were used at concentrations ranging from 10^4^ to 2 × 10^5^ nanoparticles/mL. Horizontal aura orange lines indicate the concentrations of exogenous extracellular vesicles at which maximal adhesion-altering effects were observed. Each dot is an independent biological replicate. Data are presented as the mean ± SD (*n* = 5). *, *P < *0.05; **, *P < *0.01; ***, *P < *0.005; ****, *P ≤ *0.0001, using nonparametric Kruskal-Wallis one-way analysis of variance with *post hoc* uncorrected Dunn’s multiple-comparison test.

We explored the interaction of these *Candida* species in biofilm mixture experiments. Combinations of equal concentrations of cells from two species were applied to a biofilm substrate and incubated for 90 min. The relative adherence of each species was determined using quantitative PCR (qPCR) with species-specific primers. Nine of the 10 species combinations resulted in a change in the adherence of either one or both species (Fig. 2A and B; also see the supplemental methods, Table S2). For these pairs, at least one of the two species exhibited enhanced adherence, compared to monospecies biofilm initiation. For one-half of these interactions, the second species adhered to a significantly lower degree, relative to single-species adhesion. For a minority of combinations (3), the comparative adhesion was enhanced for both species. Interestingly, two species common to these interactions are C. auris and C. tropicalis.

10.1128/mbio.02988-22.2 TABLE S2 Primer sequences utilized for quantitative mixed-*Candida* species experiments. Download Table S2, PDF file, 0.1 MB. Copyright © 2022 Zarnowski et al. 2022 Zarnowski et al. https://creativecommons.org/licenses/by/4.0/ This content is distributed under the terms of the Creative Commons Attribution 4.0 International license.

### Impact of exogenous cross-species vesicles on biofilm initiation.

We next examined the role of vesicles in this interspecies interaction during biofilm initiation by combining wild-type vesicles and cells from different species and then quantifying adhering cells. Biofilm extracellular vesicles from each *Candida* species were collected and added to the biofilm adhesion assay for each species, over a concentration range relevant to the adhesion time period, in a checkerboard format. The presence of intraspecies extracellular vesicles during the adhesion phase markedly enhanced the adherent burden of organisms more than 100% (Fig. 2C; also see Fig. S3 and the supplemental methods). Conversely, addition of vesicles from disparate species most often reduced biofilm initiation or exhibited no effect on this infection phase.

10.1128/mbio.02988-22.5 FIG S3 Effects of exogenous *Candida* biofilm extracellular vesicles on biofilm adhesion of reference *Candida* species. Biofilm cultures of reference strains were amended with wild-type extracellular vesicles isolated from five different *Candida* species biofilm culture supernatants. Lines represent the mean of 8 technical replicates, whereas marquis orange arrows indicate the concentrations of exogenous extracellular vesicles added during biofilm seeding at which maximal adhesion-altering effects were observed. Data are presented as the mean ± SD (*n* = 5). *, *P < *0.05; **, *P < *0.01; ***, *P < *0.005; ****, *P ≤ *0.0001, using nonparametric Kruskal-Wallis one-way analysis of variance with *post hoc* uncorrected Dunn’s multiple-comparison test. CA, Candida albicans; CT, Candida tropicalis; CP, Candida parapsilosis; CG, Candida glabrata; CR, Candida auris. Download FIG S3, PDF file, 0.3 MB. Copyright © 2022 Zarnowski et al. 2022 Zarnowski et al. https://creativecommons.org/licenses/by/4.0/ This content is distributed under the terms of the Creative Commons Attribution 4.0 International license.

### Conclusions.

These data help disentangle the microbial interactions and underlying mechanisms utilized by *Candida* species to establish biofilms and survive in the biofilm environment. Cells in these communities undergo a variety of physiologic changes during biofilm growth. Microbes are not limited to a single kind of interaction. We find both mutualistic and antagonistic symbiotic interactions among strains within the *Candida* genus, depending on the phase of biofilm formation. Cargo delivered by extracellular vesicles contributes to interactions across the biofilm life span. The current studies found that vesicles from *Candida* species favor the origin species but provide a means of reducing adhesive attributes of other species in the genus. Interspecies competition often is the result of competition for nutrients. We observed an interference competition among *Candida* species. Similar interactions among bacterial species, with one organism producing antibiotics that kill competing species, have been described (27). The current observations are unique, in that extracellular vesicle cargo contributes to a reduction in *Candida* biofilm initiation. Future studies exploring the mechanism of this competitive interaction may provide pharmacologic targets or strategies to prevent *Candida* biofilm formation.

### Data availability.

The complete data set is presented in the text and the supplemental material.

## References

[1] Hall-Stoodley L, Costerton JW, Stoodley P. 2004. Bacterial biofilms: from the natural environment to infectious diseases. Nat Rev Microbiol 2:95–108. doi:10.1038/nrmicro821. 15040259

[2] Chandra J, Kuhn DM, Mukherjee PK, Hoyer LL, McCormick T, Ghannoum MA. 2001. Biofilm formation by the fungal pathogen *Candida albicans*: development, architecture, and drug resistance. J Bacteriol 183:5385–5394. doi:10.1128/JB.183.18.5385-5394.2001. 11514524 PMC95423

[3] Costerton JW, Stewart PS, Greenberg EP. 1999. Bacterial biofilms: a common cause of persistent infections. Science 284:1318–1322. doi:10.1126/science.284.5418.1318. 10334980

[4] Uppuluri P, Chaturvedi AK, Srinivasan A, Banerjee M, Ramasubramaniam AK, Kohler JR, Kadosh D, Lopez-Ribot JL. 2010. Dispersion as an important step in the *Candida albicans* biofilm developmental cycle. PLoS Pathog 6:e1000828. doi:10.1371/journal.ppat.1000828. 20360962 PMC2847914

[5] Rodrigues ME, Gomes F, Rodrigues CF. 2019. *Candida* spp./bacteria mixed biofilms. J Fungi (Basel) 6:5. doi:10.3390/jof6010005. 31861858 PMC7151131

[6] Harriott MM, Noverr MC. 2011. Importance of *Candida*-bacterial polymicrobial biofilms in disease. Trends Microbiol 19:557–563. doi:10.1016/j.tim.2011.07.004. 21855346 PMC3205277

[7] Hogan DA, Kolter R. 2002. *Pseudomonas*-*Candida* interactions: an ecological role for virulence factors. Science 296:2229–2232. doi:10.1126/science.1070784. 12077418

[8] Lohse MB, Gulati M, Johnson AD, Nobile CJ. 2018. Development and regulation of single- and multi-species *Candida albicans* biofilms. Nat Rev Microbiol 16:19–31. doi:10.1038/nrmicro.2017.107. 29062072 PMC5726514

[9] Drescher K, Nadell CD, Stone HA, Wingreen NS, Bassler BL. 2014. Solutions to the public goods dilemma in bacterial biofilms. Curr Biol 24:50–55. doi:10.1016/j.cub.2013.10.030. 24332540 PMC3935403

[10] Nadell CD, Drescher K, Foster KR. 2016. Spatial structure, cooperation and competition in biofilms. Nat Rev Microbiol 14:589–600. doi:10.1038/nrmicro.2016.84. 27452230

[11] Schluter J, Nadell CD, Bassler BL, Foster KR. 2015. Adhesion as a weapon in microbial competition. ISME J 9:139–149. doi:10.1038/ismej.2014.174. 25290505 PMC4268496

[12] Yan J, Nadell CD, Stone HA, Wingreen NS, Bassler BL. 2017. Extracellular-matrix-mediated osmotic pressure drives *Vibrio cholerae* biofilm expansion and cheater exclusion. Nat Commun 8:327. doi:10.1038/s41467-017-00401-1. 28835649 PMC5569112

[13] Hibbing ME, Fuqua C, Parsek MR, Peterson SB. 2010. Bacterial competition: surviving and thriving in the microbial jungle. Nat Rev Microbiol 8:15–25. doi:10.1038/nrmicro2259. 19946288 PMC2879262

[14] Kong EF, Tsui C, Kucharikova S, Andes D, Van Dijck P, Jabra-Rizk MA. 2016. Commensal protection of *Staphylococcus aureus* against antimicrobials by *Candida albicans* biofilm matrix. mBio 7:e01365-16. doi:10.1128/mBio.01365-16. 27729510 PMC5061872

[15] Duerkop BA, Varga J, Chandler JR, Peterson SB, Herman JP, Churchill ME, Parsek MR, Nierman WC, Greenberg EP. 2009. Quorum-sensing control of antibiotic synthesis in *Burkholderia thailandensis*. J Bacteriol 191:3909–3918. doi:10.1128/JB.00200-09. 19376863 PMC2698390

[16] Brown L, Wolf JM, Prados-Rosales R, Casadevall A. 2015. Through the wall: extracellular vesicles in Gram-positive bacteria, mycobacteria and fungi. Nat Rev Microbiol 13:620–630. doi:10.1038/nrmicro3480. 26324094 PMC4860279

[17] Zaborowski MP, Balaj L, Breakefield XO, Lai CP. 2015. Extracellular vesicles: composition, biological relevance, and methods of study. Bioscience 65:783–797. doi:10.1093/biosci/biv084. 26955082 PMC4776721

[18] Albuquerque PC, Nakayasu ES, Rodrigues ML, Frases S, Casadevall A, Zancope-Oliveira RM, Almeida IC, Nosanchuk JD. 2008. Vesicular transport in *Histoplasma capsulatum*: an effective mechanism for trans-cell wall transfer of proteins and lipids in ascomycetes. Cell Microbiol 10:1695–1710. doi:10.1111/j.1462-5822.2008.01160.x. 18419773 PMC2562661

[19] Bielska E, Sisquella MA, Aldeieg M, Birch C, O'Donoghue EJ, May RC. 2018. Pathogen-derived extracellular vesicles mediate virulence in the fatal human pathogen *Cryptococcus gattii*. Nat Commun 9:1556. doi:10.1038/s41467-018-03991-6. 29674675 PMC5908794

[20] Coelho C, Brown L, Maryam M, Vij R, Smith DFQ, Burnet MC, Kyle JE, Heyman HM, Ramirez J, Prados-Rosales R, Lauvau G, Nakayasu ES, Brady NR, Hamacher-Brady A, Coppens I, Casadevall A. 2019. *Listeria monocytogenes* virulence factors, including listeriolysin O, are secreted in biologically active extracellular vesicles. J Biol Chem 294:1202–1217. doi:10.1074/jbc.RA118.006472. 30504226 PMC6349127

[21] Zarnowski R, Noll A, Chevrette MG, Sanchez H, Jones R, Anhalt H, Fossen J, Jaromin A, Currie C, Nett JE, Mitchell A, Andes DR. 2021. Coordination of fungal biofilm development by extracellular vesicle cargo. Nat Commun 12:6235. doi:10.1038/s41467-021-26525-z. 34716343 PMC8556236

[22] Zarnowski R, Sanchez H, Covelli AS, Dominguez E, Jaromin A, Bernhardt J, Mitchell KF, Heiss C, Azadi P, Mitchell A, Andes DR. 2018. *Candida albicans* biofilm-induced vesicles confer drug resistance through matrix biogenesis. PLoS Biol 16:e2006872. doi:10.1371/journal.pbio.2006872. 30296253 PMC6209495

[23] Zarnowski R, Sanchez H, Jaromin A, Zarnowska UJ, Nett JE, Mitchell AP, Andes D. 2022. A common vesicle proteome drives fungal biofilm development. Proc Natl Acad Sci USA 119:e2211424119. doi:10.1073/pnas.2211424119. 36095193 PMC9501958

[24] Horton MV, Johnson CJ, Kernien JF, Patel TD, Lam BC, Cheong JZA, Meudt JJ, Shanmuganayagam D, Kalan LR, Nett JE. 2020. *Candida auris* forms high-burden biofilms in skin niche conditions and on porcine skin. mSphere 5:e00910-19. doi:10.1128/mSphere.00910-19. 31969479 PMC6977180

[25] Proctor DM, Dangana T, Sexton DJ, Fukuda C, Yelin RD, Stanley M, Bell PB, Baskaran S, Deming C, Chen Q, Conlan S, Park M, NISC Comparative Sequencing Program, Welsh RM, Vallabhaneni S, Chiller T, Forsberg K, Black SR, Pacilli M, Kong HH, Lin MY, Schoeny ME, Litvintseva AP, Segre JA, Hayden MK. 2021. Integrated genomic, epidemiologic investigation of *Candida auris* skin colonization in a skilled nursing facility. Nat Med 27:1401–1409. doi:10.1038/s41591-021-01383-w. 34155414 PMC9396956

[26] Sexton DJ, Bentz ML, Welsh RM, Derado G, Furin W, Rose LJ, Noble-Wang J, Pacilli M, McPherson TD, Black S, Kemble SK, Herzegh O, Ahmad A, Forsberg K, Jackson B, Litvintseva AP. 2021. Positive correlation between *Candida auris* skin-colonization burden and environmental contamination at a ventilator-capable skilled nursing facility in Chicago. Clin Infect Dis 73:1142–1148. doi:10.1093/cid/ciab327. 33978150 PMC8492228

[27] Li Z, Clarke AJ, Beveridge TJ. 1998. Gram-negative bacteria produce membrane vesicles which are capable of killing other bacteria. J Bacteriol 180:5478–5483. doi:10.1128/JB.180.20.5478-5483.1998. 9765585 PMC107602

