# Supplementary material for: Extracellular Vesicles Contribute to Mixed-Fungal Species Competition during Biofilm Initiation
Source: mBio. 2022 Nov 15;13(6):e02988-22. doi: 10.1128/mbio.02988-22 (PMC9765065; doi:10.1128/mbio.02988-22)
Supplement: TABLE S1 [file mbio.02988-22-s0005.pdf]

**Table S1.** *Candida* strain genotypes used in this study

| Strain/Gene | Phenotype | Genetic makeup* | Reference |
|---|---|---|---|
| ***Candida albicans*** | | | |
| SN152 | reference strain<br>His$^-$Leu$^-$Arg$^-$ | *his1Δ/his1Δ*; *leu2Δ/leu2Δ*; *arg4Δ/arg4Δ*<br>*URA3/ura3Δ::imm*$^{434}$; *IRO1/iro1Δ::imm*$^{434}$ | (1) |
| SN250 | reference strain<br>homozygote<br>Arg | *his1Δ/his1Δ*; *leu2Δ::CD HIS1/leu2Δ::CM LEU2*; arg4Δ/arg4Δ<br>*URA3/ura3Δ::imm*$^{434}$; *IRO1/iro1Δ::imm*$^{434}$ | (2) |
| URZ585 | *cht3* homozygote<br>Arg$^-$ | *his1Δ/his1Δ*; *leu2Δ/leu2Δ*; *arg4Δ/arg4Δ*<br>*URA3/ura3Δ::imm*$^{434}$; *IRO1/iro1Δ::imm*$^{434}$*; cht3Δ::CD HIS1/cht3Δ::CM LEU2* | (3) |
| ***Candida tropicalis*** | | | |
| CAY2597 | reference strain | *C. tropicalis* wild type strain | (4) |
| CAY3764 | reference strain<br>His$^-$Leu$^-$ | *his1Δ::FRT/his1Δ::FRT, leu2Δ::FRT/leu2Δ::FRT* | (4) |
| URZ922 | *cht3* homozygote<br>prototroph | *his1Δ::FRT/his1Δ::FRT, leu2Δ::FRT/leu2Δ::FRT, cht3::CM LEU2/cht3::CD HIS1* | (5) |
| URZ978 | *cht3* complement<br>prototroph/Nat1$^+$ | *his1Δ::FRT/his1Δ::FRT, leu2Δ::FRT/leu2Δ::FRT, cht3::CM LEU2/cht3::CD HIS1,CM leu2::CHT3-NAT1* | (5) |
| URZ928 | *mp65* homozygote<br>prototroph | *his1Δ::FRT/his1Δ::FRT, leu2Δ::FRT/leu2Δ::FRT, tos1::CM LEU2/tos1::CD HIS1* | (5) |
| URZ982 | *mp65* complement<br>prototroph/Nat1$^+$ | *his1Δ::FRT/his1Δ::FRT, leu2Δ::FRT/leu2Δ::FRT, tos1::CM LEU2/tos1::CD HIS1,CM leu2::TOS1-NAT1* | (5) |
| URZ933 | *sun41* homozygote<br>prototroph | *his1Δ::FRT/his1Δ::FRT, leu2Δ::FRT/leu2Δ::FRT, sun41::CM LEU2/sun41::CD HIS1* | (5) |
| URZ984 | *sun41* complement<br>prototroph/Nat1$^+$ | *his1Δ::FRT/his1Δ::FRT, leu2Δ::FRT/leu2Δ::FRT, sun41::CM LEU2/sun41::CD HIS1,CM leu2::SUN41-NAT1* | (5) |
| URZ925 | *tos1* homozygote<br>prototroph | *his1Δ::FRT/his1Δ::FRT, leu2Δ::FRT/leu2Δ::FRT, tos1::CM LEU2/tos1::CD HIS1* | (5) |
| URZ980 | *tos1* complement<br>prototroph/Nat1$^+$ | *his1Δ::FRT/his1Δ::FRT, leu2Δ::FRT/leu2Δ::FRT, tos1::CM LEU2/tos1::CD HIS1,CM leu2::TOS1-NAT1* | (5) |
| URZ937 | *zrt2* homozygote<br>prototroph | *his1Δ::FRT/his1Δ::FRT, leu2Δ::FRT/leu2Δ::FRT, zrt2::CM LEU2/zrt2::CD HIS1* | (5) |
| URZ986 | *zrt2* complement<br>prototroph/Nat1$^+$ | *his1Δ::FRT/his1Δ::FRT, leu2Δ::FRT/leu2Δ::FRT, zrt2::CM LEU2/zrt2::CD HIS1,CM leu2::ZRT2-NAT1* | (5) |
| ***Candida parapsilosis*** | | | |
| CLIB214 | reference strain | *C. parapsilosis* wild type strain | (6) |
| CPL2H1 | reference strain<br>His$^-$Leu$^-$ | *leu2Δ::FRT/leu2Δ::FRT, his1Δ::FRT/his1Δ::FRT* | (6) |
| URZ904 | *cht3* homozygote<br>prototroph | *leu2Δ::FRT/leu2Δ::FRT, his1Δ::FRT/his1Δ::FRT, cht3::CM LEU2/cht3::CD HIS1* | (5) |
| URZ992 | *cht3* complement<br>prototroph/Nat1$^+$ | *leu2Δ::FRT/leu2Δ::FRT, his1Δ::FRT/his1Δ::FRT, cht3::CM LEU2/cht3::CD HIS1, CM leu2::CHT3-NAT1* | (5) |
| URZ913 | *mp65* homozygote<br>prototroph | *leu2Δ::FRT/leu2Δ::FRT, his1Δ::FRT/his1Δ::FRT, mp65::CM LEU2/mp65::CD HIS1* | (5) |
| URZ999 | *mp65* complement<br>prototroph/Nat1$^+$ | *leu2Δ::FRT/leu2Δ::FRT, his1Δ::FRT/his1Δ::FRT, mp65::CM LEU2/mp65::CD HIS1, CM leu2::MP65-NAT1* | (5) |
| URZ911 | *sun41* homozygote<br>prototroph | *leu2Δ::FRT/leu2Δ::FRT, his1Δ::FRT/his1Δ::FRT, sun41::CM LEU2/sun41::CD HIS1* | (5) |
| URZ908 | *tos1* homozygote<br>prototroph | *leu2Δ::FRT/leu2Δ::FRT, his1Δ::FRT/his1Δ::FRT, tos1::CM LEU2/tos1::CD HIS1* | (5) |
| URZ996 | *tos1* complement<br>prototroph/Nat1$^+$ | *leu2Δ::FRT/leu2Δ::FRT, his1Δ::FRT/his1Δ::FRT, tos1::CM LEU2/tos1::CD HIS1, CM leu2::TOS1-NAT1* | (5) |
| URZ917 | *zrt2* homozygote<br>prototroph | *leu2Δ::FRT/leu2Δ::FRT, his1Δ::FRT/his1Δ::FRT, zrt2::CM LEU2/zrt2::CD HIS1* | (5) |
| URZ002 | *zrt2* complement<br>prototroph/Nat1$^+$ | *leu2Δ::FRT/leu2Δ::FRT, his1Δ::FRT/his1Δ::FRT, zrt2::CM LEU2/zrt2::CD HIS1, CM leu2::ZRT2-NAT1* | (5) |
| ***Candida glabrata*** | | | |
| ATCC2001 | reference strain | *C. glabrata* wild type strain | (7) |
| HTL | reference strain<br>His$^-$Trp$^-$ | *his3*Δ::FRT, *leu2*Δ::FRT, *trp1*Δ::FRT | (8) |
| URZ953 | *cht3* homozygote<br>Trp$^-$/Nat1$^+$ | *his3*Δ::FRT, *leu2*Δ::FRT, *trp1*Δ::FRT, *cht3*Δ::NAT1 | (5) |
| URZ964 | *cht3* complement<br>Trp$^-$/HygB$^+$ | *his3*Δ::FRT, *leu2*Δ::FRT, *trp1*Δ::FRT, *cht3*Δ::NAT1/NAT1::CHT3-HygB | (5) |

| | | | |
|---|---|---|---|
| URZ959 | *mp65* homozygote Trp$^-$/Nat1$^+$ | *his3*Δ::FRT, *leu2*Δ::FRT, *trp1*Δ::FRT, *mp65*Δ::NAT1 | (5) |
| URZ969 | *mp65* complement Trp$^-$/HygB$^+$ | *his3*Δ::FRT, *leu2*Δ::FRT, *trp1*Δ::FRT, *mp65*Δ::NAT1/NAT1::MP65-HygB | (5) |
| URZ957 | *tos1* homozygote Trp$^-$/Nat1$^+$ | *his3*Δ::FRT, *leu2*Δ::FRT, *trp1*Δ::FRT, *tos1*Δ::NAT1 | (5) |
| URZ965 | *tos1* complement Trp$^-$/HygB$^+$ | *his3*Δ::FRT, *leu2*Δ::FRT, *trp1*Δ::FRT, *tos1*Δ::NAT1/NAT1::TOS1-HygB | (5) |
| URZ962 | *zrt2* homozygote Trp$^-$/Nat1$^+$ | *his3*Δ::FRT, *leu2*Δ::FRT, *trp1*Δ::FRT, *zrt2*Δ::NAT1 | (5) |
| URZ973 | *zrt2* complement Trp$^-$/HygB$^+$ | *his3*Δ::FRT, *leu2*Δ::FRT, *trp1*Δ::FRT, *zrt2*Δ::NAT1/NAT1::ZRT2-HygB | (5) |
| ***Candida auris*** | | | |
| B11804 | Reference strain | *Candida auris* wild type Colombian isolate of the South American clade IV | (9) |
| URZ034 | *cht3* homozygote Nat1$^+$ | *cht3*Δ::NAT1 | (5) |
| URZ017 | *cht3* complement HygB$^+$ | *cht3*Δ::NAT1/NAT1::CHT3-HygB | (5) |
| URZ036 | *mp65* homozygote Nat1$^+$ | *mp65*Δ::NAT1 | (5) |
| URZ013 | *mp65* complement HygB$^+$ | *mp65*Δ::NAT1/NAT1::MP65-HygB | (5) |
| URZ038 | *sun41* homozygote Nat1$^+$ | *sun41*Δ::NAT1 | (5) |
| URZ015 | *sun41* complement HygB$^+$ | *sun41*Δ::NAT1/NAT1::SUN41-HygB | (5) |
| URZ040 | *tos1* homozygote Nat1$^+$ | *tos1*Δ::NAT1 | (5) |
| URZ011 | *tos1* complement HygB$^+$ | *tos1*Δ::NAT1/NAT1::TOS1-HygB | (5) |
| URZ042 | *zrt2* homozygote Nat1$^+$ | *zrt2*Δ::NAT1 | (5) |
| URZ021 | *zrt2* complement HygB$^+$ | *zrt2*Δ::NAT1/NAT1::ZRT2-HygB | (5) |

** CD – Candida dubliniensis; CM – Candida maltosa*


1. Hall-Stoodley L, Costerton JW, & Stoodley P (2004) Bacterial biofilms: from the natural environment to infectious diseases. *Nat Rev Microbiol* 2(2):95-108.
2. Chandra J*, et al.* (2001) Biofilm formation by the fungal pathogen Candida albicans: development, architecture, and drug resistance. *J Bacteriol* 183(18):5385-5394.
3. Costerton JW, Stewart PS, & Greenberg EP (1999) Bacterial biofilms: a common cause of persistent infections. *Science* 284(5418):1318-1322.
4. Uppuluri P*, et al.* (2010) Dispersion as an important step in the Candida albicans biofilm developmental cycle. *PLoS Pathog* 6(3):e1000828.
5. Rodrigues ME, Gomes F, & Rodrigues CF (2019) Candida spp./Bacteria Mixed Biofilms. *J Fungi (Basel)* 6(1).
6. Harriott MM & Noverr MC (2011) Importance of Candida-bacterial polymicrobial biofilms in disease. *Trends Microbiol* 19(11):557-563.
7. Hogan DA & Kolter R (2002) Pseudomonas-Candida interactions: an ecological role for virulence factors. *Science* 296(5576):2229-2232.
8. Lohse MB, Gulati M, Johnson AD, & Nobile CJ (2018) Development and regulation of single- and multi-species Candida albicans biofilms. *Nat Rev Microbiol* 16(1):19-31.
9. Drescher K, Nadell CD, Stone HA, Wingreen NS, & Bassler BL (2014) Solutions to the public goods dilemma in bacterial biofilms. *Curr Biol* 24(1):50-55.
10. Nadell CD, Drescher K, & Foster KR (2016) Spatial structure, cooperation and competition in biofilms. *Nat Rev Microbiol* 14(9):589-600.

11. Schluter J, Nadell CD, Bassler BL, & Foster KR (2015) Adhesion as a weapon in microbial competition. *ISME J* 9(1):139-149.
12. Yan J, Nadell CD, Stone HA, Wingreen NS, & Bassler BL (2017) Extracellular-matrix-mediated osmotic pressure drives Vibrio cholerae biofilm expansion and cheater exclusion. *Nat Commun* 8(1):327.
13. Hibbing ME, Fuqua C, Parsek MR, & Peterson SB (2010) Bacterial competition: surviving and thriving in the microbial jungle. *Nat Rev Microbiol* 8(1):15-25.
14. Kong EF*, et al.* (2016) Commensal Protection of Staphylococcus aureus against Antimicrobials by Candida albicans Biofilm Matrix. *mBio* 7(5).
15. Duerkop BA*, et al.* (2009) Quorum-sensing control of antibiotic synthesis in Burkholderia thailandensis. *J Bacteriol* 191(12):3909-3918.
16. Brown L, Wolf JM, Prados-Rosales R, & Casadevall A (2015) Through the wall: extracellular vesicles in Gram-positive bacteria, mycobacteria and fungi. *Nat Rev Microbiol* 13(10):620-630.
17. Zaborowski MP, Balaj L, Breakefield XO, & Lai CP (2015) Extracellular Vesicles: Composition, Biological Relevance, and Methods of Study. *Bioscience* 65(8):783-797.
18. Albuquerque PC*, et al.* (2008) Vesicular transport in Histoplasma capsulatum: an effective mechanism for trans-cell wall transfer of proteins and lipids in ascomycetes. *Cell Microbiol* 10(8):1695-1710.
19. Bielska E*, et al.* (2018) Pathogen-derived extracellular vesicles mediate virulence in the fatal human pathogen Cryptococcus gattii. *Nat Commun* 9(1):1556.
20. Coelho C*, et al.* (2019) Listeria monocytogenes virulence factors, including listeriolysin O, are secreted in biologically active extracellular vesicles. *J Biol Chem* 294(4):1202-1217.
21. Zarnowski R*, et al.* (2021) Coordination of fungal biofilm development by extracellular vesicle cargo. *Nat Commun* 12(1):6235.
22. Zarnowski R*, et al.* (2018) Candida albicans biofilm-induced vesicles confer drug resistance through matrix biogenesis. *PLoS Biol* 16(10):e2006872.
23. Zarnowski R*, et al.* (2022) A common vesicle proteome drives fungal biofilm development. *Proc Natl Acad Sci U S A* 119(38):e2211424119.
24. Horton MV*, et al.* (2020) Candida auris Forms High-Burden Biofilms in Skin Niche Conditions and on Porcine Skin. *mSphere* 5(1).
25. Proctor DM*, et al.* (2021) Integrated genomic, epidemiologic investigation of Candida auris skin colonization in a skilled nursing facility. *Nat Med* 27(8):1401-1409.
26. Sexton DJ*, et al.* (2021) Positive Correlation Between Candida auris Skin-Colonization Burden and Environmental Contamination at a Ventilator-Capable Skilled Nursing Facility in Chicago. *Clin Infect Dis* 73(7):1142-1148.
27. Li Z, Clarke AJ, & Beveridge TJ (1998) Gram-negative bacteria produce membrane vesicles which are capable of killing other bacteria. *J Bacteriol* 180(20):5478-5483.
28. Moore GE, Gerner RE, & Franklin HA (1967) Culture of normal human leukocytes. *JAMA* 199(8):519-524.
29. Shannon P*, et al.* (2003) Cytoscape: a software environment for integrated models of biomolecular interaction networks. *Genome Res* 13(11):2498-2504.
30. Gardiner C, Ferreira YJ, Dragovic RA, Redman CW, & Sargent IL (2013) Extracellular vesicle sizing and enumeration by nanoparticle tracking analysis. *J Extracell Vesicles* 2.
31. Noble SM & Johnson AD (2005) Strains and strategies for large-scale gene deletion studies of the diploid human fungal pathogen Candida albicans. *Eukaryot Cell* 4(2):298-309.
32. Noble SM, French S, Kohn LA, Chen V, & Johnson AD (2010) Systematic screens of a Candida albicans homozygous deletion library decouple morphogenetic switching and pathogenicity. *Nat Genet* 42(7):590-598.

33. Mancera E, Porman AM, Cuomo CA, Bennett RJ, & Johnson AD (2015) Finding a Missing Gene: EFG1 Regulates Morphogenesis in Candida tropicalis. *G3 (Bethesda, Md.)* 5(5):849-856.

34. Holland LM*, et al.* (2014) Comparative phenotypic analysis of the major fungal pathogens Candida parapsilosis and Candida albicans. *PLoS Pathog* 10(9):e1004365.

35. Anonymous (1996) *Resource Sharing in Biomedical Research*, eds Berns KI, Bond EC, & Manning FJWashington (DC)).

36. Schwarzmuller T*, et al.* (2014) Systematic phenotyping of a large-scale Candida glabrata deletion collection reveals novel antifungal tolerance genes. *PLoS Pathog* 10(6):e1004211.

37. Dominguez E*, et al.* (2018) Conservation and Divergence in the Candida Species Biofilm Matrix Mannan-Glucan Complex Structure, Function, and Genetic Control. *mBio* 9(2).

38. Guiver M, Levi K, & Oppenheim BA (2001) Rapid identification of candida species by TaqMan PCR. *J Clin Pathol* 54(5):362-366.

39. Lima A, Widen R, Vestal G, Uy D, & Silbert S (2019) A TaqMan Probe-Based Real-Time PCR Assay for the Rapid Identification of the Emerging Multidrug-Resistant Pathogen Candida auris on the BD Max System. *J Clin Microbiol* 57(7).
