# Supplementary material for: Extracellular Vesicles Contribute to Mixed-Fungal Species Competition during Biofilm Initiation
Source: mBio. 2022 Nov 15;13(6):e02988-22. doi: 10.1128/mbio.02988-22 (PMC9765065; doi:10.1128/mbio.02988-22)
Supplement: TEXT S1 [file mbio.02988-22-s0004.pdf]

Supplemental Information

Materials and Methods

Fungal strains, microbiological media and culture conditions

Candida strains used in this study along with their genotypes are listed in **Table S1**. Strains were stored in 15% glycerol frozen at -80°C and routinely maintained on YPD agar plates (1% yeast extract, 2% Bacto™ peptone, 2% dextrose, 2% Bacto™ agar). Liquid planktonic cultures were grown in broth YPD (1% yeast extract, 2% Bacto™ peptone, 2% dextrose) rotating at 200 rpm at 30°C. For biofilm assays, strains were cultured in filter-sterilized Roswell Park Memorial Institute medium 1640 (RPMI), buffered with 4-morpholinepropanesulfonic acid (MOPS) and pH adjusted to 7.0 (1).

Biofilm adhesion assay

Ninety-six-well flat-bottom polystyrene plates were used to assess biofilm adhesion (2). Fungal cell inocula (10^6 cells/ml) were prepared out of overnight yeast cultures in YPD at 30°C, followed by dilution in RPMI-MOPS based on count numbers with an automated Countess™ II cell counter (Invitrogen). One hundred μl of yeast cells per well were seeded. Plates were incubated 90 min at 37°C, the growth medium was then removed and non-adherent cells were gently washed out with PBS. The density of adhered fungal cells was determined by the XTT assay as described above. Adhesion capacity of biofilms was calculated using the change in absorbance compared to that of controls.

Exogenous extracellular vesicle addback assays and functional network construction

Biological impact of exogenous extracellular vesicles on Candida biofilm adhesion was determined in 96-well plates. Both reference wild type and CHT3 null deletion mutants were used in this assay. Fungal cell inocula (10^6 cells/ml) were prepared out of overnight yeast cultures in YPD at 30°C, followed by dilution in RPMI-MOPS based on count numbers with the automated Countess™ II cell counter (Invitrogen). One hundred μl of yeast cells per well were seeded. Extracellular vesicles isolated from all five tested Candida species were used in combinations in biofilms of all five Candida species at normalized concentrations ranging between 1×10^4 and 3×10^6 particles/ml. Exogenous extracellular vesicles were applied during biofilm inoculation. Biofilms growth in cultures with and without exogenous extracellular vesicles was evaluated by the XTT assay (2). The obtained phenotypic outcomes were organized into a visual Candida biofilm phenotypic network using the Cytoscape platform (3).

Dual Candida species adhesion assay

Adhesion of dual Candida species biofilms was assessed in 96-well plates. Fungal cell inocula (10^6 cells/ml) were prepared out of overnight yeast cultures in YPD at 30°C, followed by dilution in RPMI-MOPS based on count numbers with the automated Countess™ II cell counter (Invitrogen). We initially tested two different seeding options (either 5×10^4 cells/species/well or 1×10^5 cells/species/well), but we concluded that the first concentration choice inculated into a limited well surface area (~0.32 cm^2) provided more accurate results. Thus, a total of one hundred μl of yeast cells (50 μl of each species) per well were seeded. Cells were mixed right before seeding and plates were then incubated for 90 min at 37°C, the growth medium was removed,

and non-adherent cells were gently wash out with PBS. Next, 100 μl of 20 mM NaOH was added to each well and fungal biofilm cells were lysed for 20 min at 100°C (2). To determine the abundance of individual Candida species, quantitative real-time PCR (qRT-PCR) was performed as follows: 20 μl reactions were set up in triplicate in 96 well plates, using 0.5 μl of isolated DNA aliquots per reaction. Real time primer-probe sets sequences (IDT) used for the assay are listed in **Table S2**. Reactions were run using the PrimeTime® Gene Expression Master Mix (IDT) per manufacturer's recommendations. Standard curves were generated with genomic DNA isolated from 10^8 cells from tested strains isolated with the MasterPure™ DNA Yeast Isolation Kit (Lucigen), and serially diluted in PCR-grade water. Cell concentration for standard curves was quantified prior to isolation with the Countess II Automated Cell Counter (Invitrogen). Real time cycles were as follows: one 3 min step at 95°C, followed by 40 cycles of 95°C for 15 s, and then 60 s at 60°C with read step. qRT-PCR was run on the BioRAD CFX96 Real Time PCR system (BioRad), and analysis was performed using CFX Maestro 2.3 software (BioRad). The obtained phenotypic outcomes were then organized into a visual Candida biofilm adhesion network using the Cytoscape platform (3).

Extracellular vesicle isolation and analysis

Candida biofilms were grown using a large-scale rolling bottle biofilm model system. Culture media were carefully decanted from the polystyrene bottles after 24 and 48 h of incubation at 37°C. Culture supernatants were filter sterilized and concentrated down to about 25 ml using a Vivaflow 200 unit (Sartorius AG) equipped with a Hydrosart 30 kDa cut-off membrane. Samples were centrifuged to remove smaller cellular debris particulates first at 10,000×g for 1 h at 4°C. The pellet was discarded, and the resulting supernatant was centrifuged again at 100,000×g for 1.5 h at 4°C. Next, the supernatant was discarded, and the pellet was then washed in 5 ml of PBS and re-centrifuged at 100,000×g for 1 h at 4°C. The collected extracellular vesicles were next polished by flash size-exclusion chromatography on a qEV/35 nm column (Izon Science), filter sterilized and stored until further use at 4°C (4).

Exosomes were quantified using nanoparticle tracking analysis (NTA). EV samples were diluted in PBS to a final volume of 1 ml and pretested to obtain an ideal 30-100 particles per frame rate using a NanoSight NS300 system coupled with an autosampler (Malvern). The following settings were applied: camera level was increased to 16 and camera gain to 2 until tested images were optimized and nanoparticles were distinctly visible without exceeding particle signal saturation. Each measurement consisted of five 1-min videos with a delay of 5 s between sample introduction and the start of the first measurement. For detection threshold analysis the counts were limited to 10-100 red crosses and no more than 5-7 blue crosses. Acquired data were analyzed using the NanoSight Software NTA 3.4 Build 3.4.003. At least 1000 events in total was tracked per sample in order to minimize data skewing based on single large particles (5).

Assessment of extracellular vesicle production in biofilms during adhesion

Quantitative analysis of EVs produced during adhesion of Candida biofilms was determined at in 96-well plates. Fungal cell inocula (10^6 cells/ml) were prepared out of overnight yeast cultures in YPD at 30°C, followed by dilution in RPMI-MOPS based on count numbers with the automated Countess™ II cell counter (Invitrogen). One hundred μl of yeast cells per well were seeded and incubated at 37°C. Supernatant samples were collected after 90 min of incubation, filter sterilized and subjected to NTA-based extracellular vesicle analysis as described above (**Fig S1**).

Statistics

Statistically significant outliers were identified based on the Grubbs' test. Data sets of equal of different sample sizes were analyzed using the nonparametric Kruskal-Wallis one-way analysis of variance with uncorrected Dunn's multiple comparisons. Data were processed with GraphPad Prism 9 for Windows 64-bit (version 9.3.1 (471)).

1. Moore GE, Gerner RE, Franklin HA. 1967. Culture of normal human leukocytes. JAMA 199:519-24.
2. Zarnowski R, Noll A, Chevrette MG, Sanchez H, Jones R, Anhalt H, Fossen J, Jaromin A, Currie C, Nett JE, Mitchell A, Andes DR. 2021. Coordination of fungal biofilm development by extracellular vesicle cargo. Nat Commun 12:6235.
3. Shannon P, Markiel A, Ozier O, Baliga NS, Wang JT, Ramage D, Amin N, Schwikowski B, Ideker T. 2003. Cytoscape: a software environment for integrated models of biomolecular interaction networks. Genome Res 13:2498-504.
4. Zarnowski R, Sanchez H, Jaromin A, Zarnowska UJ, Nett JE, Mitchell AP, Andes D. 2022. A common vesicle proteome drives fungal biofilm development. Proc Natl Acad Sci U S A 119:e2211424119.
5. Gardiner C, Ferreira YJ, Dragovic RA, Redman CW, Sargent IL. 2013. Extracellular vesicle sizing and enumeration by nanoparticle tracking analysis. J Extracell Vesicles 2.
