# Supplementary material for: Extracellular Vesicles Contribute to Mixed-Fungal Species Competition during Biofilm Initiation
Source: mBio. 2022 Nov 15;13(6):e02988-22. doi: 10.1128/mbio.02988-22 (PMC9765065; doi:10.1128/mbio.02988-22)
Supplement: FIG S1 [file mbio.02988-22-s0001.pdf]

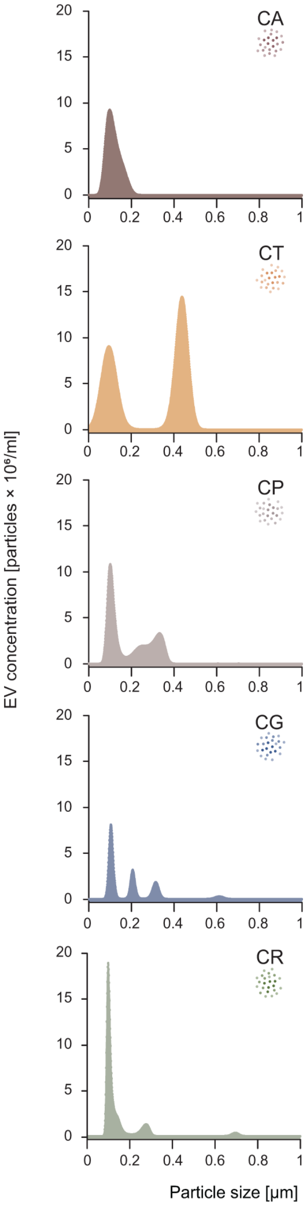

Fig S1. Concentration of Candida biofilm EVs during early biofilm formation. EVs were isolated from the tested reference strains at 90-min followed by NTA analysis of filter-sterilized culture supernatants. Both qualitative and quantitative profiles of EVs quickly undergo dramatic changes shifting from highly convoluted heterogeneous mixtures towards less complex and more abundant exosome-like nanoparticle amalgamates. Data are presented as the mean, $n = 3$. CA – *Candida albicans*; CT – *Candida tropicalis*; CP – *Candida parapsilosis*; CG – *Candida glabrata*; CR – *Candida auris*.
