# Supplementary material for: Extracellular Vesicles Contribute to Mixed-Fungal Species Competition during Biofilm Initiation
Source: mBio. 2022 Nov 15;13(6):e02988-22. doi: 10.1128/mbio.02988-22 (PMC9765065; doi:10.1128/mbio.02988-22)
Supplement: FIG S2 [file mbio.02988-22-s0002.pdf]

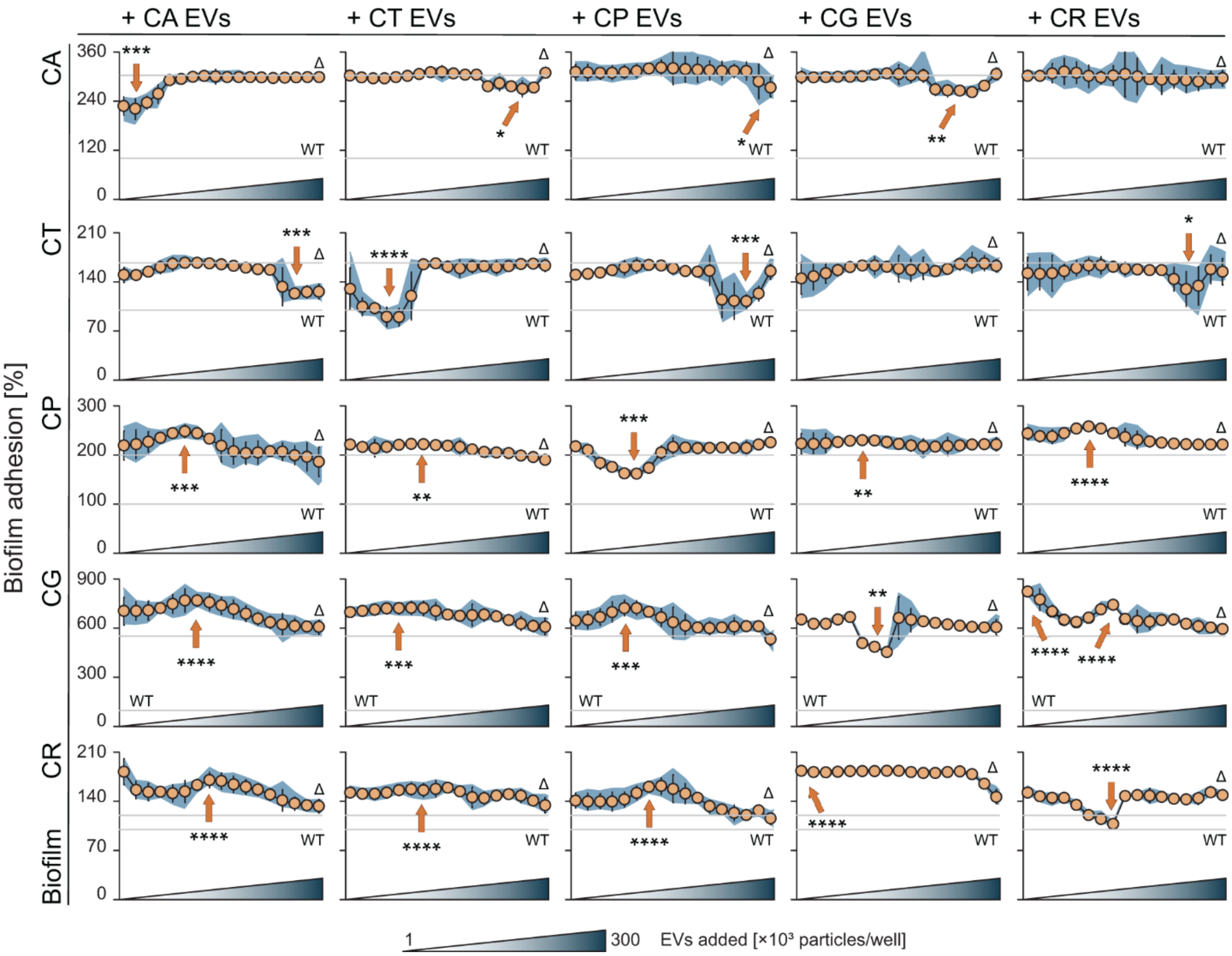

Fig S2. Effects of exogenous Candida biofilm EVs on biofilm adhesion of CHT3 null mutants. Biofilm cultures of adhesion-altered mutant strains (grouped in rows) were amended with WT EVs (columns) isolated from five different Candida species biofilm culture supernatants. Lines represent the mean of 8 technical replicates and the shaded blue area represents minimal and maximal value range distribution. Marquis orange arrows indicate concentrations of exogenous EVs added during biofilm seeding, at which maximal adhesion-alteration effects were observed. Data are presented as the mean ± SD; $n = 5$; *$P<0.05$; **$P<0.01$; ***$P<0.005$*; ****$P \leq 0.0001$, using non-parametric Kruskal–Wallis one-way analysis of variance with post hoc uncorrected Dunn's multiple comparison test. CA – *Candida albicans*; CT – *Candida tropicalis*; CP – *Candida parapsilosis*; CG – *Candida glabrata*; CR – *Candida auris*.
