# Supplementary material for: Extracellular Vesicles Contribute to Mixed-Fungal Species Competition during Biofilm Initiation
Source: mBio. 2022 Nov 15;13(6):e02988-22. doi: 10.1128/mbio.02988-22 (PMC9765065; doi:10.1128/mbio.02988-22)
Supplement: TABLE S2 [file mbio.02988-22-s0006.pdf]

**Table S2.** PCR primers used in this study

| Target Candida Species | Primer type | Sequence |
|---|---|---|
| *Candida albicans*(1) | forward | GGGTTTGCTTGAAAGACGGTA |
| | reverse | TTGAAGATATACGTGGTGGACGTTA |
| | probe | 6-FAM-ACCTAAGCC/ZEN/ATTGTCAAAGCGATCCCG/IABkFQ |
| *Candida tropicalis*(1) | forward | CGTGGTAACTTATTTTAAGCG |
| | reverse | GCTTAAGTTCAGCGGGTAGTCCTA |
| | probe | 6FAM-TGGCCACCA/ZEN/TTTATTTCATAACTTTGACC/IABkFQ |
| *Candida parapsilosis*(1) | forward | GGGTTTGGTGTTGAGCGATAC |
| | reverse | GGAGTTTGTACCAATGAGTGGAAA |
| | probe | 6FAM-CTCCGCCTT/ZEN/TCTTTCAAGCAAACCCAG/IABkFQ |
| *Candida glabrata*(1) | forward | TTTCTCCTGCCTGCGCTTAA |
| | reverse | ACGCACACTCCCAGGTCTTT |
| | probe | 6-FAM-AGAACACCC/ZEN/ACCAACCGCGCA/IABkFQ |
| *Candida auris*(2) | forward | CGTGATGTCTTCTCACCAATCT |
| | reverse | TACCTGATTTGAGGCGACAAC |
| | probe | 6-FAM/TTTGTGAAT/ZEN/GCAACGCCACCGC/IABkFQ |


1. Guiver M, Levi K, Oppenheim BA. 2001. Rapid identification of candida species by TaqMan PCR. J Clin Pathol 54:362-6.
2. Lima A, Widen R, Vestal G, Uy D, Silbert S. 2019. A TaqMan Probe-Based Real-Time PCR Assay for the Rapid Identification of the Emerging Multidrug-Resistant Pathogen Candida auris on the BD Max System. J Clin Microbiol 57.
