# Supplementary material for: Extracellular Vesicles Contribute to Mixed-Fungal Species Competition during Biofilm Initiation
Source: mBio. 2022 Nov 15;13(6):e02988-22. doi: 10.1128/mbio.02988-22 (PMC9765065; doi:10.1128/mbio.02988-22)
Supplement: FIG S3 [file mbio.02988-22-s0003.pdf]

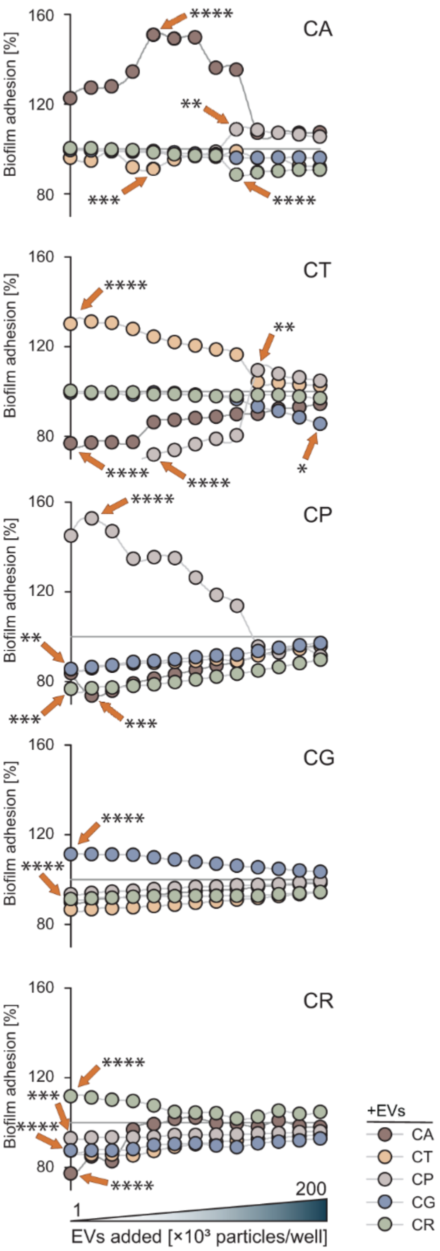

Fig S3. Effects of exogenous Candida biofilm EVs on biofilm adhesion of reference Candida species. Biofilm cultures of reference strains were amended with WT EVs isolated from five different Candida species biofilm culture supernatants. Lines represent the mean of 8 technical replicates, whereas marquis orange arrows indicate concentrations of exogenous EVs added during biofilm seeding, at which maximal adhesion-alteration effects were observed. Data are presented as the mean $\pm$ SD; n = 5; *P<0.05; **P<0.01; ***P<0.005*; ****$P \leq 0.0001$, using non-parametric Kruskal–Wallis one-way analysis of variance with post hoc uncorrected Dunn's multiple comparison test. CA – *Candida albicans*; CT – *Candida tropicalis*; CP – *Candida parapsilosis*; CG – *Candida glabrata*; CR – *Candida auris*.
